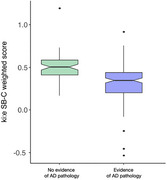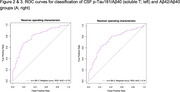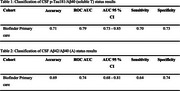# Discriminating AD Biomarker Groups Based on a Digital Speech Biomarker for Cognition (SB‐C)

**DOI:** 10.1002/alz70856_097031

**Published:** 2025-12-24

**Authors:** Alexandra König, Elisa Mallick, Johannes Tröger, Nicklas Linz, Anika Wuestefeld, Erik Stomrud, Pontus Tideman, Oskar Hansson, Sebastian Palmqvist

**Affiliations:** ^1^ ki elements UG, Saarbrücken, Germany; ^2^ Côte d'Azur University, Nice, NA, France; ^3^ ki:elements GmbH, Saarbrücken, Germany; ^4^ Clinical Memory Research Unit, Department of Clinical Sciences Malmö, Lund University, Lund, Sweden; ^5^ Memory Clinic, Skåne University Hospital, Malmö, Skåne, Sweden; ^6^ Clinical Memory Research Unit, Department of Clinical Sciences Malmö, Faculty of Medicine, Lund University, Sweden, Lund, Sweden; ^7^ Lund University, Lund, Sweden; ^8^ Clinical Memory Research Unit, Department of Clinical Sciences, Lund University, and Memory Clinic, Skåne University Hospital, Malmö, Sweden; ^9^ Clinical Memory Research Unit, Department of Clinical Sciences Malmö, Faculty of Medicine, Lund University, Lund, Sweden

## Abstract

**Background:**

This study investigates the potential of speech analysis, using a digital Speech Biomarker for Cognition (SB‐C), to non‐invasively discriminate between Alzheimer's disease (AD) biomarker groups, specifically amyloid‐β (Aβ) and *p*‐tau status.

**Methods:**

Data were obtained from the Swedish BioFINDER‐Primary Care study, which includes patients undergoing evaluation for cognitive symptoms in primary care. Participants with subjective cognitive decline (SCD) or mild cognitive impairment (MCI) and available cerebrospinal fluid (CSF) Alzheimer's disease (AD) biomarkers were included.

SB‐C scores, including subscores (executive function, memory, and processing speed), were extracted from Semantic Verbal Fluency (SVF) and RBANS List Learning task recordings using ki:elements’ proprietary speech analysis pipeline. Optimal SB‐C score cut‐offs to differentiate AD pathology (presence/absence) were determined using an independent separate cohort.

These cut‐offs were validated then on the Swedish BioFINDER‐Primary Care cohort to discriminate between CSF Aβ42/Aβ40 (A+/‐) and CSF *p*‐Tau181/Aβ40 (soluble T+/‐, where Aβ40 acts as a reference protein), with classification performance assessed through sensitivity, specificity, balanced accuracy, and ROC‐AUC metrics.

**Results:**

Data were collected from 241 participants from which 79 showed no evidence of AD pathology (mean [SD]age:71.2[8]; 43 women) and 162 showed evidence of AD pathology (mean [SD]age:77.6[6.28]; 95 women). The cutoff analysis showed optimal cut‐offs of the SB‐C normed z‐scores of 0.43 to discriminate presence of AD pathology versus no AD pathology (see Figure 1).

The area under the ROC curve (AUC) was 0.79 for the SB‐C score using CSF *p*‐Tau181/Aβ40 as outcome. With the optimized cut‐offs the accuracy was 71% (sensitivity = 70%, specificity = 73%). For CSF Aβ42/Aβ40 positivity/negativity classification performance was at 69% accuracy (sens = .64, spec = .74) and the AUC at 0.74.

**Conclusion:**

The SB‐C demonstrates potential as a non‐invasive and scalable tool for discriminating between presence and absence of AD pathology, particularly in primary care settings. By leveraging automated speech analysis, it offers a practical approach to enhance screening efficiency, reduce costs, and minimize patient burden.